# Representing, learning, and controlling complex object interactions

**DOI:** 10.1007/s10514-018-9740-7

**Published:** 2018-04-30

**Authors:** Yilun Zhou, Benjamin Burchfiel, George Konidaris

**Affiliations:** 1 Computer Science and Artificial Intelligence Lab, Massachusetts Institute of Technology, Cambridge, USA; 2 Duke Robotics, Duke University, Durham, USA; 3 Department of Computer Science, Brown University, Providence, USA

**Keywords:** Robotics, Task representation, Task learning, Markov decision process

## Abstract

We present a framework for representing scenarios with complex object interactions, where a robot cannot directly interact with the object it wishes to control and must instead influence it via intermediate objects. For instance, a robot learning to drive a car can only change the car’s pose indirectly via the steering wheel, and must represent and reason about the relationship between its own grippers and the steering wheel, and the relationship between the steering wheel and the car. We formalize these interactions as chains and graphs of Markov decision processes (MDPs) and show how such models can be learned from data. We also consider how they can be controlled given known or learned dynamics. We show that our complex model can be collapsed into a single MDP and solved to find an optimal policy for the combined system. Since the resulting MDP may be very large, we also introduce a planning algorithm that efficiently produces a potentially suboptimal policy. We apply these models to two systems in which a robot uses learning from demonstration to achieve indirect control: playing a computer game using a joystick, and using a hot water dispenser to heat a cup of water.

## Introduction

As robots become more physically capable of interacting with the world by manipulating the objects in it, their applications will demand more powerful representations of the effects of those interactions. Such representations will be essential for robots to determine how to act in the world in order to achieve their goals. While there has been a great deal of research on reasoning about directly manipulating objects—for example opening a door (Meeussen et al. 2010) or grasping and folding a towel (Maitin-Shepard et al. 2010)—many real-world situations are more complex. Often, the object a robot directly manipulates is not the object of interest; the robot must use one object to indirectly affect the state of some other object(s).

Consider teaching a robot to drive a car. The robot’s direct interaction with the environment is via the steering wheel. In this task, we are not trying to teach the robot to move the steering wheel to a specific position, or even to follow a specific steering-wheel trajectory. Instead, we are using the steering wheel to control the car. Although controlling the car is the primary objective of our actions, that can only be achieved though interaction with an intermediate object (the steering wheel). The robot cannot learn only the interaction with the intermediate object (because that ignores the state of the car), but we cannot ignore it either (because it is the only way to control the car).

A representation that is aware of this interaction structure would facilitate learning in at least two ways. First, learning each part of the interaction could be done independently, in an appropriate state space: when the robot learns how its grippers rotate the steering wheel, it need not care about the car position; when it learns how the wheel controls the car, it need not even be in the driver’s seat (observing a human driver is sufficient). Second, learned knowledge can be transferred even if parts of the task change: if the robot must now steer a ship, it need only focus on the interaction between the steering wheel and the ship, without having to re-learn how to rotate the wheel. This transfer of knowledge could substantially reduce the time required to learn everyday tasks.

We therefore introduce a framework for representing such complex interactions as chains of Markov decision processes and show how such a chain can be learned from data. We then extend the framework to handle more general object interaction graphs, which express interaction relationships that may change over time. For example, a robot may pour cold water from a bottle into an electric kettle, boil the water, and then pour the water back into the bottle. In the first stage of the task, the robot uses the bottle to affect the state of the kettle (i.e. amount of water inside), but in the final stage, it uses the kettle to affect the state of the bottle. To facilitate this, we introduce the use of *activation classifiers* and *deactivation classifiers* as representations of the circumstances under which an interaction between two objects becomes active or inactive. We also consider how these models can be controlled given known or learned dynamics. We show that our complex model can be collapsed into a single MDP and solved to find an optimal policy for the combined system. Since the resulting MDP may be very large, we also introduce a planning algorithm that efficiently produces a potentially suboptimal policy. We use our framework to create robot systems that use learning by demonstration to operate a computer game using a joystick, and re-sequence learned skills to operate a hot water dispenser to warm a cup of cold water.^1^

## Background

Control learning problems are often modeled as Markov decision processes (MDPs) (Sutton and Barto 1998) which can be described by a tuple $\{S, A, T, R\}$ where *S* is a set of (possibly continuous) states, *A* is a set of (possibly continuous) actions, $T: S\times A \times S \rightarrow [0,1]$ is a transition function that yields probability of an action causing a transition from one state to another, and $R: S\times A \times S \subseteq \mathbb {R}$ is a reward function that maps a transition to a real-valued reward. A solution to an MDP takes the form of a policy $\pi :S\times A\rightarrow [0,1]$ that returns the probability of taking a particular action in a given state, in order to maximize the *return* (expected sum of discounted reward):

1 $$\begin{aligned} {{\mathrm{arg\,max}}}_\pi \mathbb E_\pi \left[ \sum _{t=0}^\infty \gamma ^t r_t\right] , \end{aligned}$$

where $0 < \gamma \le 1$ is a discount factor expressing a preference for immediate over delayed reward. If the agent has a model of *T* and *R* it can generate a policy by planning; if it does not, it must learn one by taking exploratory actions in its environment.

In learning from demonstration (LfD) (Argall et al. 2009), a robot is given demonstrated trajectories obtained by executing an expert’s policy, and must be able to reproduce the policy. There are multiple possible strategies here, depending on how much the robot knows about the MDP. For example, if the robot does not know the transition or reward function, it may try to learn the policy directly, using the state-action pairs in the demonstrated trajectories as labeled training examples in a supervised learning setting. If the robot knows the transition model but neither knows nor observes the reward function, it could produce a policy that tries to follow the demonstrated trajectories, or use inverse reinforcement learning (Abbeel and Ng 2004) to try to recover the reward function. If the robot is given the reward function, it may also use the state-action-reward tuples in the demonstration trajectories as labeled training data to learn the MDP transition and reward functions, and then solve the resulting MDP.

Task representation and learning in the context of both MDPs and in the specific setting of learning from data have received a great deal of attention, but when applied to robotics this is almost always in the service of directly manipulating objects of interest. To the best of our knowledge, we are the first to explicitly consider object-object interactions in the same manner as robot-object interactions.

## Interaction chains

We now present a model that captures scenarios in which a robot’s interactions with one object cause changes in the state of another; that other object may cause changes to further objects, until we reach a particular object of interest. We therefore model the complete dynamics—from the object the robot is currently manipulating, to the object of interest—as a chain (Sect. 4 generalizes our model to a graph, which can capture more complex interactions).

An interaction chain thus consists of *N* objects $O_1, O_2,\ldots , O_N$, where $O_1$ is the robot. Each $O_i$ has an associated MDP $M_i\equiv \{S_i, A_i, T_i, R_i\}$, where $S_i$ is the set of states for $O_i$, $A_i$ is the set of “actions” (explained below),

$$\begin{aligned} T_i: S_i^{(t)}\times A_{i}^{(t+1)} \times S_i^{(t+1)} \rightarrow [0,1] \end{aligned}$$

is a transition function that encodes the transition dynamics at level *i*, and

$$\begin{aligned} R_i: S_i^{(t)}\times A_{i}^{(t+1)} \times S_i^{(t+1)} \rightarrow \mathbb {R} \end{aligned}$$

is a reward function. Note that we use $A^{(t+1)}$ to denote the action resulting in the transition from $S^{(t)}$ to $S^{(t+1)}$.

In the chain model, we assume that interactions only exist between successive objects $O_{i-1}$ and $O_{i}$, for all $i\in \{2,\ldots ,N\}$. For such an interaction, we call $O_{i-1}$ the *predecessor object* and $O_i$ the *successor object*. The interactions are modeled by coupling their MDPs so that the state of object $i-1$ affects (or controls) the state of object *i*, *and thus serves as the action of* $M_i$, i.e.:

2 $$\begin{aligned} S_{i-1}^{(t)}\equiv A_i^{(t)}\,\,\,i=2,\ldots ,N. \end{aligned}$$

$S_1$ describes the state of the robot, and $A_1$ describes the motor control actions available to it. Changes in the robot state (along with the passive dynamics at each level) are ultimately the source of all state transitions in our model, as objects interact with each other through the chain. We make no particular distinction between the robot and other objects, except that the robot is the root of the chain and cannot be controlled by other objects.

**Fig. 1:**
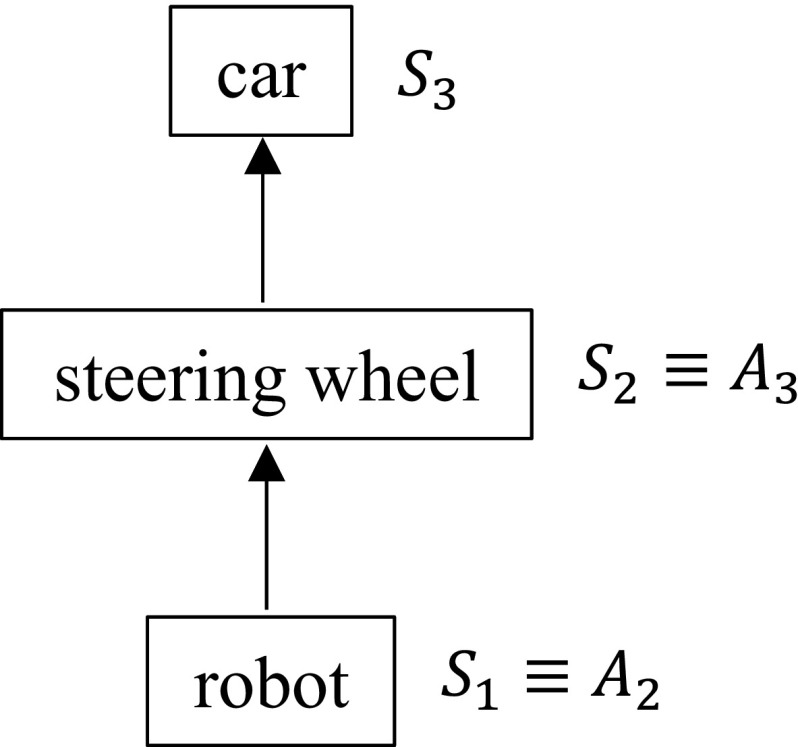
The interaction chain for the car driving example

In the car-driving task, $S_1\equiv A_2$ is the state of the robot (e.g. gripper position), $S_2\equiv A_3$ is the state of the steering wheel (e.g. rotation angle), and $S_3$ is the state of the car (e.g. pose and speed). Figure 1 shows the interaction chain for this example.

There are two reasons for using MDPs to define each primitive interaction. First, it is reasonable to assume the Markov property is present in each individual interaction. For example, the turning angle of the car only depends on the current state of the steering wheel, rather than a history of the steering wheel rotation. This assumption is common in robot manipulation models and can be expected to hold in most situations. In addition, as we will show in subsequent sections, we can construct control algorithms for an MDP-based model (which specifically rely on the Markov property holding for the transition function). With a suitably constructed state feature set, our model can fully account for dynamic effects by simply include the appropriate derivative features (e.g. velocity for a moving object).

During implementation we discovered a subtle point about timing: since most physical systems are continuous in nature but we are using discrete time, we found that when modeling the interaction between successive objects $O_{i-1}$ and $O_{i}$, as a practical matter the action causing the transition from $s_{i}^{(t)}$ to $s_{i}^{(t+1)}$ is better predicted by $s_{i-1}^{(t+1)}$ than by $s_{i-1}^{(t)}$. Recall that the state of the predecessor object serves as the action of the successor object. Thus for $O_{i-1}$ and $O_{i}$, we have $S_{i-1}\equiv A_i$. Therefore, for two consecutive time points, *t* and $t+1$, we have four states: $s_{i-1}^{(t)}$, $s_{i}^{(t)}$, $s_{i-1}^{(t+1)}$, $s_{i}^{(t+1)}$. It may seem natural to credit $s_{i-1}^{(t)}$ for making the transition in $O_i$ from $s_{i}^{(t)}$ to $s_{i}^{(t+1)}$. However, it is actually better to treat $s_{i-1}^{(t+1)}$ as the action for the transition. For example, consider the interaction between the robot and the steering wheel. When the robot holds the steering wheel, $s_{\mathrm {robot}}^{(t+1)}$, the hand position at time $t+1$, instead of $s_{\mathrm {robot}}^{(t)}$, best predicts $s_{\mathrm {wheel}}^{(t+1)}$, the steering wheel rotation angle at time $t+1$. More generally, at time *t*, it is difficult to predict the action in effect for the duration between time *t* and $t+1$, which is revealed at time $t+1$. The discreteness of our system explains why robot movement during the experiment appeared “jerky.” For continuous time MDP, we could integrate the transition function with respect to time to obtain the state evolution.

**Fig. 2:**
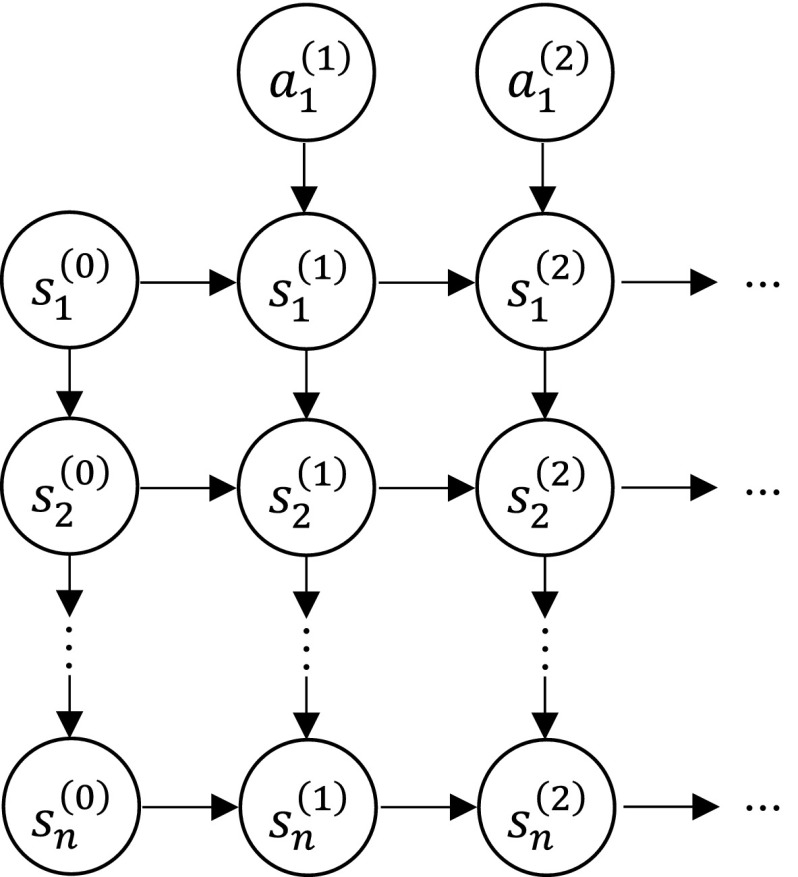
The forward transition graph for an interaction chain

Using this notation, Fig. 2 shows a state transition diagram modeling both time and object level. Nodes that do not have arrows represent initial conditions or robot actions; all others have two incoming arrows representing the two inputs to the transition function. The top row denotes the robot actions.

### Learning a model

Learning a model of a chain of MDPs involves approximating the transition functions for all individual levels. The data required for learning takes the form of transition samples from a particular level of the chain:

$$\begin{aligned} \left( s_i^{(t)}, a_i^{(t+1)}, s_i^{(t+1)}\right) , \end{aligned}$$

and can be obtained from human demonstration or by the agent itself, or as a mix of both at different levels. Learning a complete model will require sufficient data at all levels of the chain, but a key advantage here is that a model of a particular level can be retained if the levels above it, or more than one level below it, change. The same is not true for the level immediately below it: if level *i* changes, then the learned model of level $i+1$ is no longer useful, since its actions depend on the state descriptor at level *i*.

The control algorithms we use later require an *inverse transition function*, which maps a state transition pair $(s_i^{(t)}, s_i^{(t+1)})$ to an action $a_i^{(t+1)} \equiv s_{i-1}^{(t+1)}$ in the hope that this action can successfully result in the desired state transition. This function can obtained by either analytically or numerically inverting the learned (forward) transition function or by directly learning it from data. Due to non-invertibility (actions are not unique) and stochasticity (action effects are not deterministic) in some systems, the inverse transition function may have to be an approximation.

### Control

In this section, we present two methods to control a chain of MDPs. The first finds an optimal solution (policy) to the entire chain by collapsing it to a single MDP at the cost of having to solve that large MDP, while the second allows for a trajectory to be specified at a particular level (e.g., by planning solely at that level) and then controls all lower levels to attempt to follow it. This trajectory following approach, based on a feedback controller, serves as both an approximation to the optimal full MDP solution and a standalone algorithm allowing trajectory-level control.

#### Solving for the optimal policy

Given a fully specified chain of MDPs with known reward and transition functions, we wish to find an optimal policy that maximizes the expected sum of discounted total rewards accrued by the robot across all objects:

3 $$\begin{aligned} E\left[ \sum _{t=1}^{\infty }\left( \gamma ^t\sum _{i=1}^{n}r_i^{(t)}\right) \right] . \end{aligned}$$

Recall that $r_i^{(t)}$ denotes the immediate reward received by the robot at time *t* from level *i* of the composite MDP, and that the agent can only select actions at level 1.

We now show that a single, flat, MDP can be constructed from the chain of MDPs such that an optimal solution to the collapsed MDP is also an optimal solution to the chain of MDPs. Because the collapsed MDP is simply an MDP like any other, existing methods such as value iteration may be used to solve it in a straightforward fashion.

We define the state set for the collapsed MDP as the Cartesian product of the states in each level of the chain of MDPs:

$$\begin{aligned} S_* = \prod _{i=1}^{n}S_i, \end{aligned}$$

while the action space consists of only the actions from the first level of the chain of MDPs—those belonging to the robot:

$$\begin{aligned} A_* = A_1. \end{aligned}$$

To derive the transition function, we make use of a convenient property of the chain of MDPs: the transition at level *i*, $T(s_i^{(t)}, a_i^{(t+1)}, s_i^{(t+1)})$ is independent of the state of all other levels given $a_i^{(t+1)}$ and $s_i^{(t)}$. Due to this factorization in the chain of MDPs and according to Fig. 2, the transition function for the collapsed MDP is:

$$\begin{aligned}&T_*(s_*^{(t)}, a_*^{(t+1)}, s_*^{(t+1)})\\&\quad =\Pr \left( s_1^{(t+1)}, \ldots , s_n^{(t+1)}|s_1^{(t)}, \ldots , s_n^{(t)},a_1^{(t+1)}\right) \\&\quad = \Pr \left( s_1^{(t+1)}|s_1^{(t)},a_1^{(t+1)})\cdot \Pr (s_2^{(t+1)}|s_2^{(t)},s_1^{(t+1)}\right) \ldots \\&\qquad \cdot \Pr \left( s_n^{(t+1)}|s_n^{(t)},s_{n-1}^{(t+1)}\right) \\&\quad =T_1\left( s_1^{(t)}, a_1^{(t+1)}, s_1^{(t+1)}\right) \cdot \prod _{i=2}^{n}T_i\left( s_i^{(t)}, s_{i-1}^{(t+1)}, s_i^{(t+1)}\right) . \end{aligned}$$

The reward function is the sum of the reward functions at each level of the chain, naturally following the objective described in Eq. 3:

$$\begin{aligned} R_*(s_*^{(t)}, a_*^{(t+1)}, s_*^{(t+1)})&=R_1(s_1^{(t)}, a_1^{(t+1)}, s_1^{(t+1)})\\&\quad +\,\sum _{i=2}^{n}R_i(s_i^{(t)}, s_{i-1}^{(t+1)}, s_i^{(t+1)}). \end{aligned}$$

Note that because the action at level *i* corresponds to the state at level $i+1$ in the chain of MDPs, it is only necessary to explicitly consider actions at the lowest level when creating the collapsed MDP. Actions at higher levels (in the chain) are implicitly captured in the state space of the collapsed MDP. This formulation preserves the full expressiveness of the state space in the chain while only preserving actions the robot can directly execute. This removes the underactuated nature of the chain, which makes solving for an optimal policy vastly easier; value or policy iteration can be applied straightforwardly to the collapsed MDP. Because the state space, rewards, and transitions are preserved, a solution to the collapsed MDP must correspond to an optimal policy in the chain. Furthermore, because the reward and transition functions are factored by chain MDP level, it is trivial to precompute portions of the collapsed MDP that are expected to stay constant. For instance, if level *i* of the chain is expected to change frequently (perhaps it refers to a particular tool being used) but the lower levels $(1, \ldots , i-1)$ and upper levels $(i+1, \ldots , n)$ are expected to stay fixed, levels $(1, \ldots , i-1)$ and $(1+1, \ldots , n)$ can be collapsed separately and stored. When level *i* changes, the full collapsed MDP can be quickly computed from the new MDP at level *i* and the two precomputed pieces.

One drawback to the collapsed MDP representation is the high dimensionality of the joint state space it creates. However, there are some conditions on individual transition and reward functions that, if met, can reduce the joint state space by discarding some state variables. Specifically, we can remove level *i* entirely from the collapsed MDP if

$$\begin{aligned}&\forall a_i\in A_i, \forall s_i^{(t)}, s_i'^{(t)}, s_i^{(t+1)} \in S_i, \\&T_i\left( s_i^{(t)}, a_i^{(t)}, s_i^{(t+1)}\right) =T_i\left( s_i'^{(t)}, a_i^{(t)}, s_i^{(t+1)}\right) \\&R_i\left( s_i^{(t)}, a_i^{(t)}, s_i^{(t+1)}\right) =R_i\left( s_i'^{(t)}, a_i^{(t)}, s_i^{(t+1)}\right) . \end{aligned}$$

Essentially, the condition on $T_i$ mandates that the next state $s_i^{(t+1)}$ is only dependent on the action $a_i^{(t+1)}$ (and not on previous state $s_i{(t)}$), and the condition on $R_i$ mandates that reward only depend on action taken and the next state. When these two conditions are met, it is easy to see that $a_{i}$ can directly serve as an action for the $(i+1)$-th object by replacing the original $a_{i+1}\equiv s_i$. In addition, the condition on reward function ensures that regardless of the previous state, the received reward is the same.

The condition on the reward function is usually met, but satisfying the condition on transition function is rarer. One example for which the condition on transition function is met is the steering wheel in the car-driving task. The robot gripper position serves as the action for steering wheel. When we assume that the robot gripper is always holding the steering wheel, the gripper position (action for steering wheel) is sufficient to predict next steering wheel angle regardless of previous steering wheel angle. Thus, we can discard the steering wheel angle variable and directly use the gripper position as the action for the car.

However, if the previous steering wheel angle does affect next angle, then we cannot discard this level. For example, if the steering wheel can rotate multiple cycles and the angle state variable can reflect it (by having the variable going beyond the $[0, 2\pi ]$ interval), then it is not sufficient to only use gripper position to determine next state rotation angle. Instead, the transition function must make use of the previous angle to determine the correct next angle. In this case, the steering wheel state variable must be kept in the collapsed MDP.

#### Approximate solutions via successive trajectory following

Since the collapsed MDP outlined in the previous section has a state space equal to the Cartesian product of individual state spaces, solving it optimally may often be impractical. To work around this problem, we developed a trajectory following algorithm built upon a feedback controller. Specifically, this controller can control the current state $s_i^{\mathrm{(cur)}}$ of a specific object toward a goal $s_i$. Then to solve the whole problem of controlling the chain, we can first compute the optimal policy for the last object, $\pi _n(s_n, a_{n})$, with $a_{n}\equiv s_{n-1}$. From here, we turn to the trajectory-following controller and control the robot to move the $(n-1)$-th object as directed by $pi_n$.

More generally, if we can afford to solve a larger MDP, then we can collapse the last *k* MDPs, which has the action being $s_{n-k}$. Then we use the trajectory following algorithm to control the $(n-k)$-th object according to the solved optimal policy for the joint last *k* objects, $\pi _{n-1}$. In addition, this feedback controller may also be used in a standalone manner if we are given only the trajectory for the last object to follow (e.g. a path that the car needs to travel on).

Concretely, our trajectory following algorithm employs inverse transition function, $T_i^{-1}:S_i\times S_i\rightarrow S_{i-1}$, such that for the interaction between object $i-1$ and object *i*, the function returns an action $s_{i-1}^{(t+1)}$ which can cause the desired transition from $s_i^{(t)}$ to $s_{i}^{(t+1)}$ (recall the time convention discussed at the end of Sect. 3).

Note that the returned $s_{i-1}^{(t+1)}$ may be impossible to reach from the current state of object $i-1$, $s_{i-1}^{(t)}$; for instance, if the robot can only turn the steering wheel gradually but $s_{i-1}^{(t)}$ and $s_{i-1}^{(t+1)}$ represents two very different angles, the transition must be effected over a longer period of time. To solve this problem, path planning (state_plan function) is performed at each level of the composite MDP. The resulting algorithm move_one_step_toward, may be called successively to achieve trajectory following and is given in Algorithm 1.
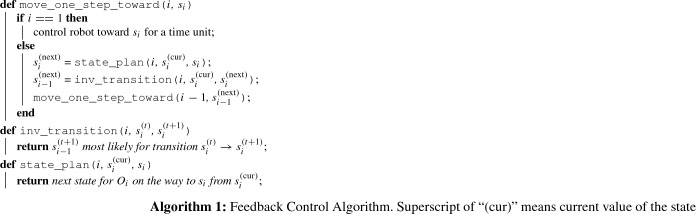
 
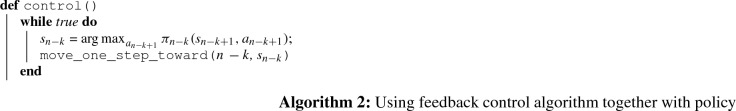


Algorithm 2 summarizes how to incorporate this controller into a solved policy $\pi _{n-k}$. The trajectory following algorithm sacrifices the optimality guarantee to gain fast computation time. However, if *individual *interactions have reasonably simple interaction transitions, then overall feedback control can yield satisfactory performance with only minor deviations from the trajectories to follow. For example, in our experiment described in Sect. 5.1, when interactions can be fitted well by polynomial functions, the trajectory is followed very closely.

In addition, the feedback control algorithm can only handle rewards for top *k* objects in the chain because while the partially collapsed MDP is solved with respect to the reward functions for the top *k* objects, the trajectory following algorithm for controlling the remaining objects are unaware of the reward function. In many cases, this constraint is acceptable since we typically care more about the behavior of objects higher up in the chain. For instance, that the car reaches the destination is more important than how steering wheels are rotated and how much energy is expended in robot actuation.

## Interaction graphs

**Fig. 3:**
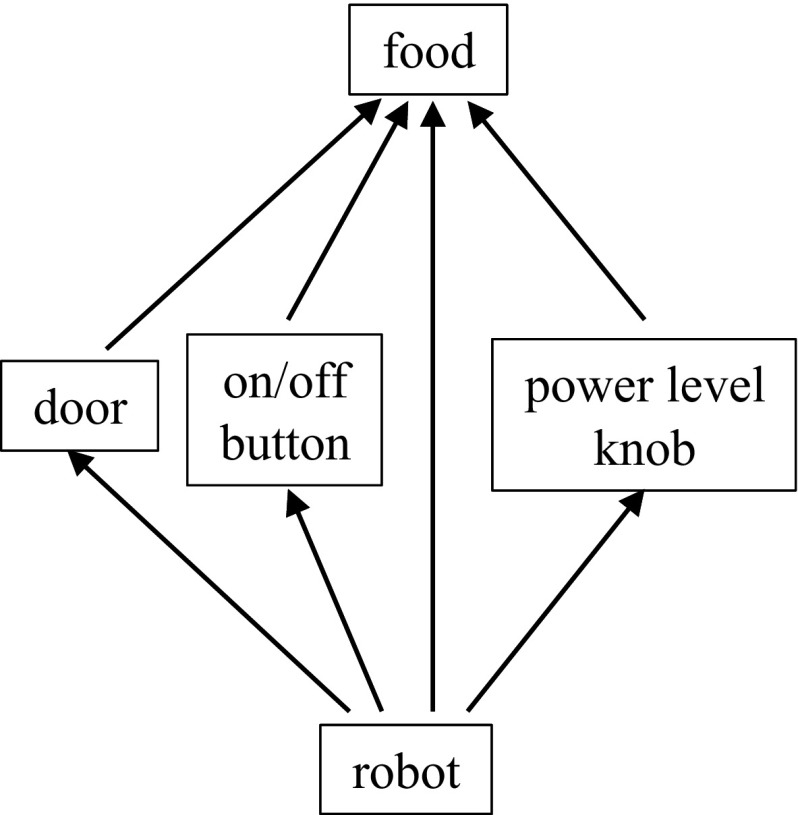
The interaction graph for the microwave oven example

The chain model presented in Sect. 3 can only describe situations in which objects are arranged in a chain, but many real-life scenarios are more complex. Consider, for example, operating a microwave oven (consisting of a door, an on/off button, and a power level knob) to heat food. The door must be open to place and retrieve food and closed for the oven to operate. The on/off button starts and stops the oven, and the power level knob changes the heating speed. All three components can influence the temperature of the food (the door influences the temperature by preventing it from changing when open), however, they do not interact with each other. Moreover, in addition to interacting with the components of the microwave, the robot can directly modify properties of the food such as its position and orientation. We must therefore extend the interaction chain to a graph where each node represents an object (a collection of state variables that can be simultaneously manipulated), and a directed edge from node *i* to node *j* represents the fact that the state of node *i* (possibly jointly with states of other nodes) *can* (but not necessarily always *does*) affect the state of node *j*. Figure 3 shows the interaction graph for the microwave oven example.

We represent an interaction graph as a collection of MDPs $M = \{M_1, \ldots , M_n\}$, along with a graph *G*(*M*, *E*) where directed edges between the MDPs represent relationships of influence. Since many objects can jointly affect one object, the action set for object *i* is now the Cartesian product of all the state sets belonging to its parents:

4 $$\begin{aligned} A_i\equiv \prod _{k:(k,i)\in E}S_k. \end{aligned}$$

When multiple objects can jointly interact with one object, the inverse transition function becomes more complex. To mitigate this problem, we distinguish between active and inactive interactions. In an interaction chain, we assume that all interactions are always “happening”; for example, as long as the robot is holding the steering wheel, any movement of the hand will induce corresponding rotation of the wheel. In an interaction graph, however, an object may only affect another in some cases. For example, the robot can only manipulate one object at a time. The states of objects that are not being manipulated progress according to their passive dynamics.

Formally, for object *i* affecting object *j*, we distinguish active transition

$$\begin{aligned} T_{i,j}: S_j^{(t)}\times S_{i}^{(t+1)} \times S_j^{(t+1)} \rightarrow [0,1] \end{aligned}$$

(note that $S_i\equiv A_j$), from inactive passive dynamics

$$\begin{aligned} T_{i,j}': S_j^{(t)}\times S_j^{(t+1)} \rightarrow [0,1], \end{aligned}$$

with the next state in the passive dynamics being purely a function of previous state.

*Activation classifiers* $\alpha$ and *deactivation classifiers* $\delta$ govern the change in interactions from inactive to active, and from active to inactive, respectively. Each classifier is a function mapping the states of the two objects into a boolean denoting (de)activating status. For interaction between object *i* and *j*,

$$\begin{aligned}&\alpha _{i,j}:S_i\times S_j \rightarrow \{0,1\},\\&\delta _{i,j}:S_i\times S_j \rightarrow \{0,1\}. \end{aligned}$$

An activation classifier returning true denotes the activation of a previously inactive interaction, and false means that the interaction remains inactive. The deactivation classifier is the reverse: returning true deactivates a currently active interaction, and false allows it to remain active. As a specific example, in the microwave oven scenario, the interaction between the robot and the food is only activated when the robot reaches and grasps the food.

### Learning

In interaction graph, the both the active and passive transition functions need to be learned for each interaction for MDP collapsing. In addition, the inverse (active) transition needs to be learned for trajectory following control. Moreover, the activation and deactivation classifiers must also be learned. To learn the interaction classifiers, the robot must be given the interaction status between two objects at consecutive time steps. It is given by hand in our example, but could in principle be learned autonomously by determining whether or not the passive dynamics of the object have been perturbed.

### Control

#### Solving for the optimal policy

For the interaction graph, in order to get a collapsed MDP whose transition function satisfies the Markov property, we need to additionally augment the state space to include all interaction activation status variables, $I_{i,j} \in \{0,1\}$ for $i,j\in E$ (recall that *E* is the set of edges of the interaction graph, representing interactions).

$$\begin{aligned} S_* = \prod _{i=1}^{n}S_i\times \prod _{(i,j)\in E}I_{i,j}. \end{aligned}$$

Then the transition function is

$$\begin{aligned} T_*=\prod _{(i,j)\in E}T^{\mathrm{true}}_{i,j}\cdot Q_{i,j} \end{aligned}$$

in which

$$\begin{aligned} T^{\mathrm{true}}_{i,j}={\left\{ \begin{array}{ll} T_{i,j}\,\,\,\quad \text{ if } \,I_{i,j}=1\\ T_{i,j}'\,\,\, \quad \text{ if } \,I_{i,j}=0 \end{array}\right. } \end{aligned}$$

selects the correct transition (active or passive) according to the activation status *I*, and

$$\begin{aligned} Q_{i,j}={\left\{ \begin{array}{ll} 1 \,\,\, \quad \text{ if }\, I_{i,j}^{(t+1)}\, \hbox {and} \,I_{i,j}^{(t)}\, \text {are consistent with}\, \alpha _{i,j}\, \hbox {and} \,\delta _{i,j}\\ 0 \,\,\, \quad \text{ otherwise } \end{array}\right. } \end{aligned}$$

is a boolean function that tests if the activation status indicators transition consistently according to activation classifier $\alpha _{i,j}$ and deactivation classifier $\delta _{i,j}$. In other words, the whole transition probability will be 0 as long as one next state activation status is not correct according to the (de)activation classifiers. The reward function is a summation of individual rewards, as before.

**Fig. 4:**
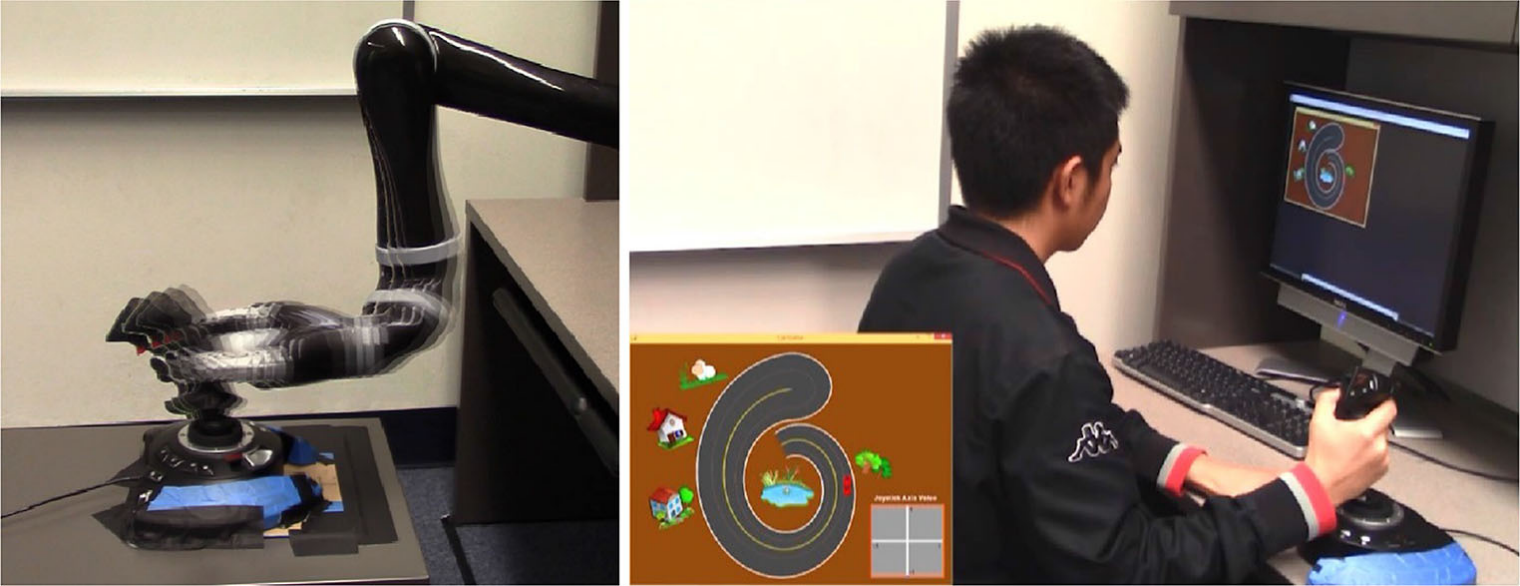
Learning at two levels. Left: learning the robot-joystick interaction by playing with the joystick. Right: learning the joystick-car interaction by observing a human playing the game (Best viewed in color)

#### Trajectory following

Trajectory following for an interaction graph is similar to that in the chain case, except that the robot must know how to activate and deactivate interactions. We model actions that perform the activation and deactivation—reaching states within the classifiers—as motor skills that can either be given by the system designer, or learned from demonstration. Specifically, from the given or learned classifiers the robot can find (and reach) states of both the manipulating and manipulated objects that will (de)activate an interaction.

With (de)activation skills, the inverse transition function should also output the which interaction it is. For example, the transition of water amount increasing in the cup has the interaction of dispensing, while the transition that the cup position changes has the interaction of robot moving the cup. The only addition to the trajectory following algorithm is that when the interaction switches (e.g. from hand moving cup to pressing a button), the previous interaction must be deactivated and the next interaction activated before the control algorithm loops.

## Experiments

We present two experiments across two different domains to demonstrate the interaction chain and graph models as applied to real-world robot tasks. In both experiments, the interaction chain/graph structure and low-level state features are known *a priori*.

### The car game

The car game domain involves a robot operating a joystick that controls a car in a custom-made video game. The interaction chain diagram was shown previously in Fig. 1, with the steering wheel being replaced by the joystick. We use a mix of autonomous learning and learning from demonstration to show that the robot can learn to control the car to follow complex trajectories.

Because of the small movements and high sensitivity of the joystick, the robot hand position is extracted directly from the robot API and the joystick configuration, also retrieved programmatically, is represented by two axis (left/right and forward/backward) values in the range of −1 to 1. The position of the car is also read programmatically.

We learn two interactions: between the robot hand and the joystick, and between the joystick and the car. We collect the data for the first interaction (robot to joystick) by having the robot directly play with the joystick and learn the association between the hand position and the joystick axis values. The robot tried about 500 hand positions to learn the relationship between the hand position and the joystick configuration. This interaction is not linear as the joystick axis values do not change linearly with tilting angle.

We used human demonstration for the second interaction (joystick to car). Both joystick angles and game states are recorded. While the game runs at 30 Hz, we could only control the robot reliably at 3 Hz: beyond this control rate, the robot would drop control commands and therefore not faithfully execute a command sequence. Thus, the training data are also collected at 3 Hz. We generated about 500 transition pairs. For both levels, the inverse transition function was learned using polynomial regression. Figure 4 shows the learning process.

**Fig. 5:**
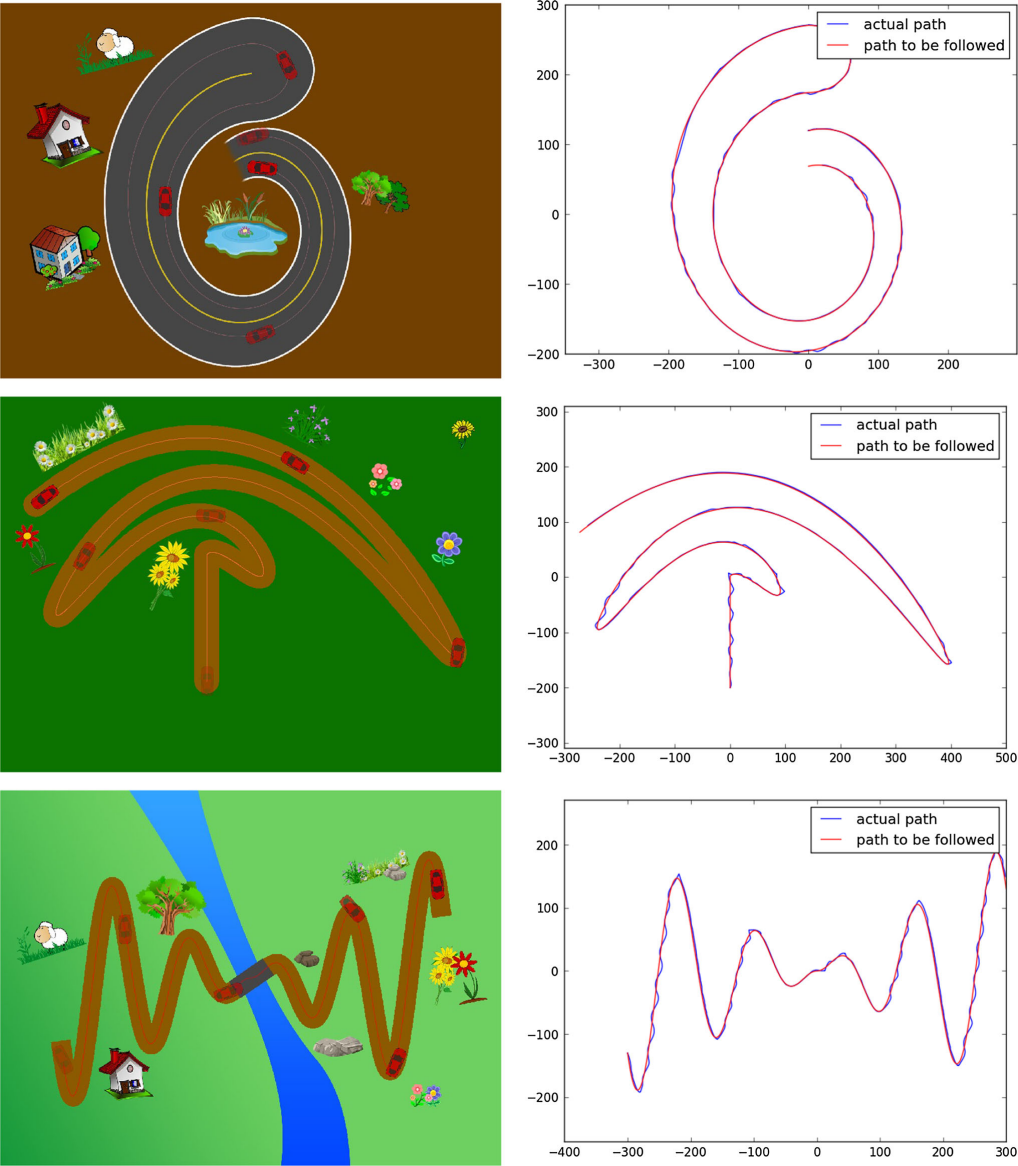
Trajectory following. Earlier car positions are more transparent. The “road” on the game interface is for visual illustration purposes only and does not constrain the motion of the car (Best viewed in color)

During execution, the robot’s goal was to follow a given car path as closely as possible. Three complex trajectories were followed by the robot. Figure 5 shows the followed trajectories overlaid on the game screen on the left, and the commanded and executed paths on the right.

We calculated the average distance between demonstrated and followed trajectories by sampling random points from the executed trajectory, finding the minimum distance of each point to the commanded trajectory (also discretized), and averaging those distances. The paths had average errors of 0.790, 1.2480, and 1.8662 pixels (on a $800\times 600$ game screen), respectively. Thus, we are able to reproduce the demonstrated trajectories very accurately on this task.

Due to the modularity of our model, it is readily adaptive to change: if the joystick position is changed, the robot need only translate its hand accordingly; if the robot gripper is changed, it need only re-learn how to hold and control the joystick; if the game is changed, the robot need only re-learn the interaction between the joystick and the new game. This occurs because, in both cases, one of the MDPs is left unchanged.

### The water dispenser

**Fig. 6:**
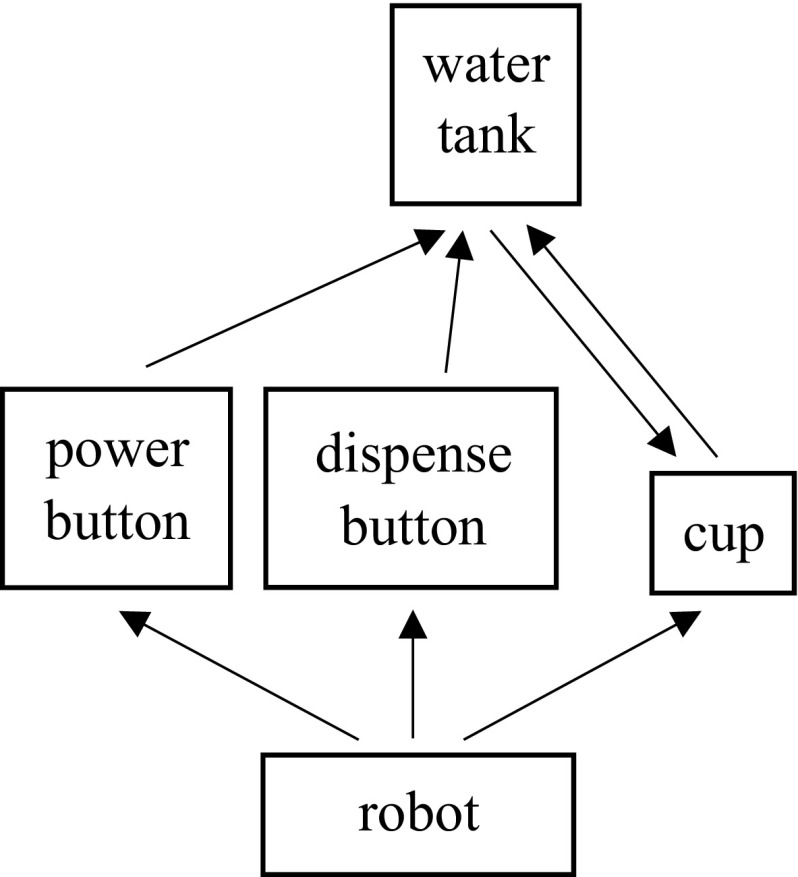
The interaction graph of the water dispenser experiment

We now demonstrate the use of an interaction graph in a system where a robot uses a hot water dispenser to turn a cup of cold water into a cup of hot water. The water dispenser has a water tank to hold water, a dispense button to dispense the water in the tank, and a power button to heat the water in the tank. Figure 6 shows the interaction graph.

We used an Intel Realsense F200 camera to track the cup pose and the depression of the power and dispense buttons. Since it is very challenging to extract information about liquid (such as the water amount in a tilted cup) and computer vision is not a focus of our work, we pre-computed the water amount for each cup tilting angle. We also estimated the water temperature in the tank during heating.

For the robot hand, the state variables included *x*, *y*, *z* coordinates in the robot frame, and the tilt angle $\theta$ necessary for pouring water from cup. We use a binary variable to model whether the fingers of the hand were open or closed. The state variables of the cup included its pose variables (defined similarly to those for the hand, but in the camera frame), and two real variables between 0 and 1 denoting the normalized amount of water (0 being empty) and the water temperature (0 being cold). Finally, it has a change in water level defined to be $\varDelta \hbox {water}\_\hbox {amount}^{(t)}\equiv \hbox {water}\_\hbox {amount}^{(t)}-\hbox {water}\_\hbox {amount}^{(t-1)}$. Only with the inclusion of this state variable can the interaction between cup and water tank be Markov. The two buttons each have a level of depression expressed as an integer between 0 and 6, since they have a maximum depression of 6 pixels in our camera setting. The water tank has three variables: water amount, water temperature, and change in water amount, defined similarly as those of the cup.

For the robot-cup interaction, activation occurs when the cup is inside the hand and the hand is closed; deactivation occurs when the hand opens. When the interaction is active, the position and tilt of the cup will follow those of hand. They are not the same, however, since they are in different reference frames. The water amount will decrease and the change in water amount will be negative if the cup is tilted. The water temperature does not change in this interaction.

For the interactions between the robot and the power/dispense button, activation occurs when the robot hand is above the button and deactivation happens at the same position. During interaction, the button level will be determined by the *z*-coordinate of the hand (its height). For both buttons, a depression greater than 3 will trigger heating and dispensing, respectively. When the dispense button is active, the water amount in the tank decreases by 1 / 16 each time unit (second) until it reaches 0, since it takes about 16 s to empty the dispenser. Similarly, when the power button is active the temperature increased by 1 / 80 until reaching 1.

Both pouring and dispensing interactions can happen between the cup and tank, albeit in different directions. The pouring interaction activates when the cup is above the tank and the dispensing interaction activates when cup is below the tank. The key to the transition function is that the water amounts *change *in opposite directions. Thus, the Markov property holds through the change in water amount. Finally, the new water temperature is an average between the old temperature and the temperature of the newly added water, weighted by their relative volumes.

**Fig. 7:**
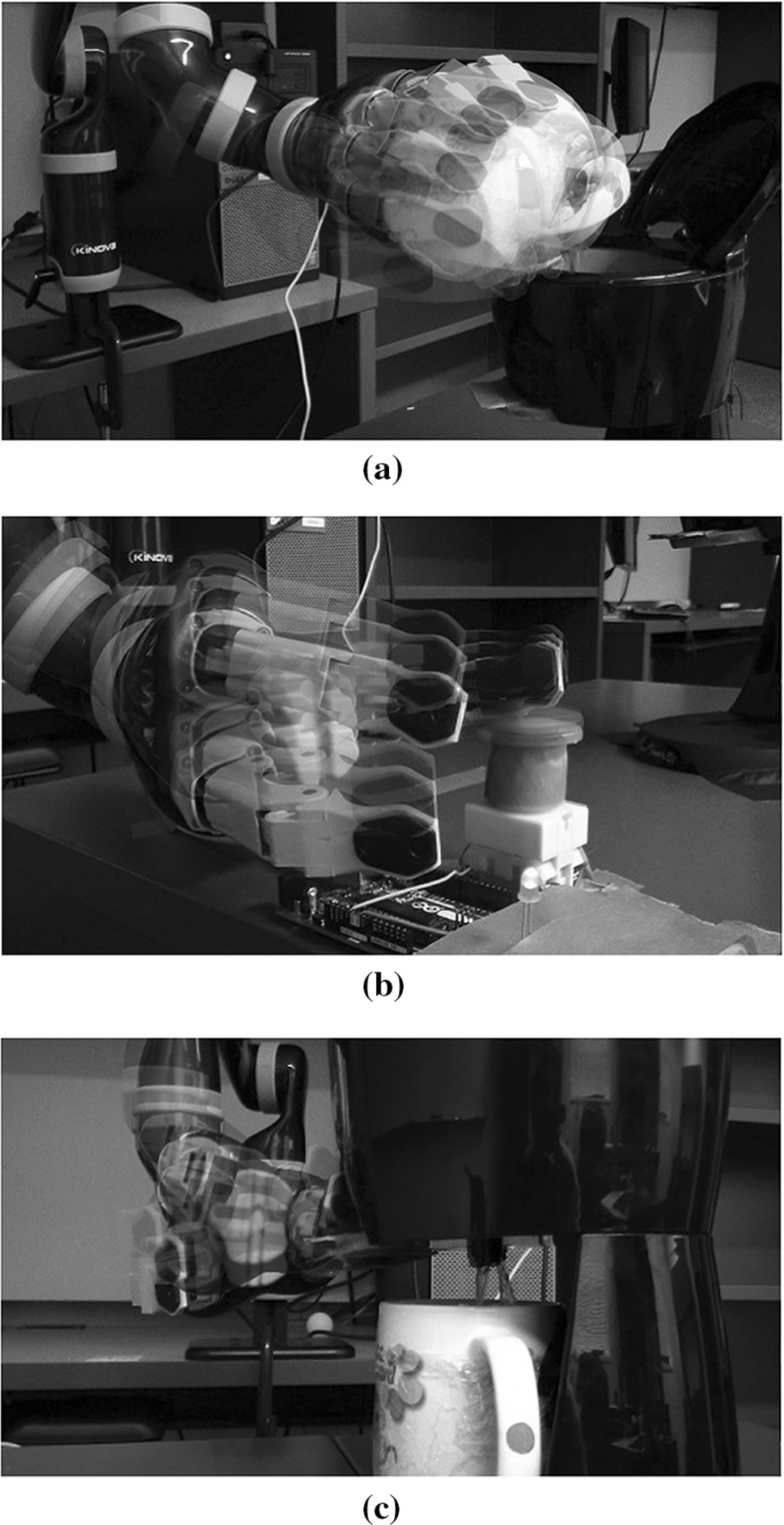
The robot learns various interactions through tele-operation (Best viewed in color). **a** Pouring water. **b** Pressing the power button. **c** Pressing the dispense button

During learning, the robot was tele-operated to complete a set of tasks that helped it learn all the interactions. Figure 7 visualizes the tele-operation. About ten demonstrations were given. It should be noted that these skill demonstrations are *unordered*. The active and inactive states are hand-labeled. From these skills, the robot learns a model of the interaction between the objects.

Given this model, the robot applied our control algorithm to obtain a cup of hot water when it is given a cup of cold water, a challenging task that requires multiple object interaction steps. Specifically, in execution, the robot must use the cup to pour the cold water into the water tank, move the cup to below the tank, press and hold the power button until the water is boiling, then press the dispense button. Once the water tank is empty, the robot must retrieve the cup of newly heated water.

**Fig. 8:**
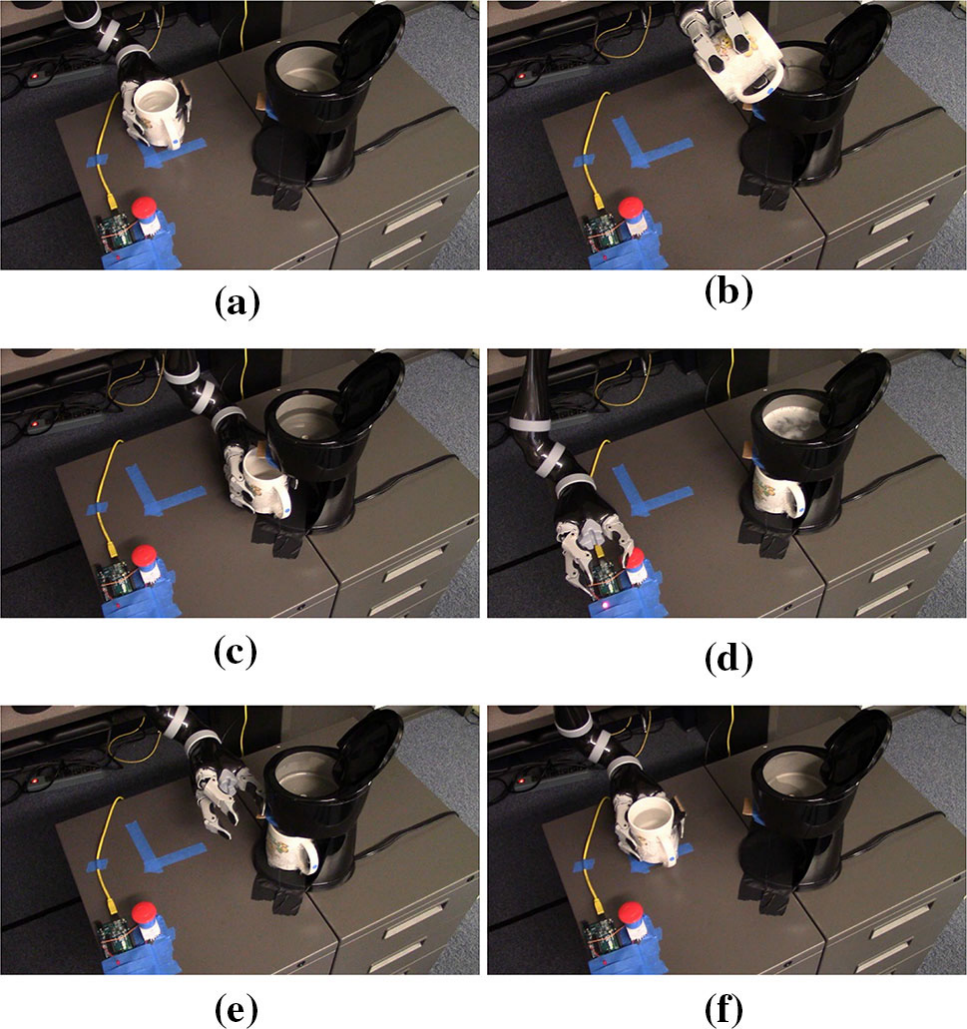
The robot autonomously sequences learned skills to heat a cup of water (Best viewed in color). **a** Pick up the cup. **b** Pour cold water to the machine. **c** Place the cup under the machine. **d** Press the power button to boil water. **e** Press the dispense button to dispense water. **f** Move the cup to original position

After we modified the control algorithm for the interaction chain model to incorporate activation and deactivation classifiers (see the Appendix), our system was able to autonomously achieve the goal using its learned knowledge. The assembled execution consists of about 1000 discrete control steps. Figure 8 shows several waypoints in the robot execution.

## Related work

There is a large body of literature on building complex skills via hierarchical learning and execution. Of particular relevance, Kolter et al. (2008) introduced hierarchical apprenticeship learning which enables the trainer to demonstrate skills at different levels of abstraction. For example, a trainer can demonstrate footstep placement on rough terrain, and also demonstrate how to do low-level motor control to locally follow the footsteps for quadruped locomotion.

In general, while hierarchical approaches are concerned with hierarchical structure that is *internal to the robot*, we are concerned with structure that exists in the relationships between objects in the world. In that sense, hierarchical approaches could be viewed as *vertical* (the robot building new skills on top of old skills), whereas our approach could be considered *horizontal* (the robot affecting control through chains of objects). For example, the layered learning approach proposed by Stone and Veloso (2000) uses a collection of machine learning components each of which either directly controls the agent’s behavior, is used as a subskill by another component, or provides input features, output features, or training examples to another such component. This structure could be considered vertical because it builds structure that is *internal to the agent* and primarily a means for combatting the complexity of generating behavior, as opposed to describing external chains of causal influence. In addition, Konidaris and Barto (2009) proposed a method to automatically connect individual actions together to make more complicated and temporally extended actions (i.e. options). This representation is also vertical in that the task hierarchy is *internal to the robot*.

In our interaction graph model, demonstrations are broken into different pieces (e.g. pressing a button, moving a cup, pouring water, etc.), and each segment can be considered a task with transition dynamics independent from state of other objects in the absence of collisions. Much work has been done on automatically breaking unstructured demonstrations into subskills (Jenkins and Matarić 2004; Kulić et al. 2009; Chiappa et al. 2009; Chiappa and Peters 2010; Grollman and Jenkins 2010; Butterfield et al. 2010; Krüger et al. 2010; Konidaris et al. 2012; Niekum et al. 2015), which could be applied in our framework to find the individual motor skills that enable or disable an interaction.

For scenarios with multiple objects, for a pick-and-place task involving multiple objects, Ekvall and Kragic (2008) identified spatio-temporal constraints from either teacher instruction or inference and reasoning over multiple demonstrations. Our activation and deactivation classifier have similar functions to the constraints in their work. In a similar vein, Konidaris et al. (2014) used classifiers to represent the conditions under which a high-level action can be executed, and used them to construct an abstract representation of a task.

## Conclusion and future work

We have introduced a flexible representation that goes beyond modeling robot-object interactions to account for object-object interactions, and showed that it can be used to learn two distinct real-life tasks involving complex object interactions.

There are several advantages of our model. First, it naturally represents interactions among objects, so that we can build complex systems capable of achieving their goals through the use of intermediate objects. In particular, tool use is an embodiment of this characteristic as tools are examples of such intermediate objects.

Second, by modeling each object as a separate MDP, we can factor the joint state space to mitigate against the curse of dimensionality. Models that are unaware of intermediate objects must represent—and therefore learn—the compound interaction of the robot monolithically. Even if individual interactions are simple, the compound one can be very hard to model.

Finally, our model can accomplish knowledge transfer. Since each interaction is represented by an MDP, the trajectory following algorithm can directly use a transferred MDP rather than learning the MDP from scratch. This step requires no overhead and directly reduces the total amount of learning, which can be very significant for low-cost robots with limited onboard computational resources, or when gathering experience is costly. Since our work does not require demonstrations to be given in the correct order, interactions can be learned at different times and from different sources. In addition, when equipped with a database of “primitive interaction models” as prior knowledge, a robot can be immediately versatile in everyday environments, without too much learning.

One limitation of the work is that the graph needs to be supplied *a priori*. Although non-expert users can come up with the graph in most cases as the graph corresponds to our common sense of causality, it would still be preferable for the robot to autonomously discover the interaction graph structure for a given task. The inclusion of prior knowledge (e.g. our interactions are causal and it is highly unlikely that a lamp lighting up will cause a button to depress), accurate sensing, and active information gathering will likely prove necessary for learning complex interaction model structures completely from scratch. In addition, ideas from structure discovery in Bayesian networks may also be relevant here.

## Footnotes

1 A video explaining the model and showing both experiments is available at https://www.youtube.com/watch?v=TJlxXX1v6S4.
